# Potential Inhibitors of SARS-CoV-2 Main Protease (M^pro^) Identified from the Library of FDA-Approved Drugs Using Molecular Docking Studies

**DOI:** 10.3390/biomedicines11010085

**Published:** 2022-12-29

**Authors:** Dipesh Kumar Verma, Srajan Kapoor, Satyajeet Das, Krishan Gopal Thakur

**Affiliations:** Structural Biology Laboratory, CSIR-Institute of Microbial Technology, Chandigarh 160036, India

**Keywords:** COVID-19, SARS-CoV-2, M^pro^, molecular docking, MM-GBSA analysis

## Abstract

The Corona Virus Infectious Disease-2019 (COVID-19) outbreak originated at Wuhan, China, in December 2019. It has already spread rapidly and caused more than 6.5 million deaths worldwide. Its causal agent is a beta-coronavirus named SARS-CoV-2. Many efforts have already been made to develop new vaccines and drugs against these viruses, but over time, it has changed its molecular nature and evolved into more lethal variants, such as Delta and Omicron. These will lead us to target its more-conserved proteins. The sequences’ BLAST and crystal structure of the main protease M^pro^ suggest a high sequence and structural conservation. M^pro^ is responsible for the proteolytic maturation of the polyprotein essential for the viral replication and transcription, which makes it an important drug target. Discovery of new drug molecules may take years before getting to the clinics. So, considering urgency, we performed molecular docking studies using FDA-approved drugs to identify molecules that could potentially bind to the substrate-binding site and inhibit SARS-CoV-2’s main protease (M^pro^). We used the Glide module in the Schrödinger software suite to perform molecular docking studies, followed by MM-GBSA-based energy calculations to score the hit molecules. Molecular docking and manual analysis suggest that several drugs may bind and potentially inhibit M^pro^. We also performed molecular simulations studies for selected compounds to evaluate protein–drug interactions. Considering bioavailability, lesser toxicity, and route of administration, some of the top-ranked drugs, including lumefantrine (antimalarial), dipyridamole (coronary vasodilator), dihydroergotamine (used for treating migraine), hexoprenaline (anti asthmatic), riboflavin (vitamin B2), and pantethine (vitamin B5) may be taken forward for further in vitro and in vivo experiments to investigate their therapeutic potential.

## 1. Introduction

Coronaviruses are a group of RNA viruses that cause diseases in mammals and birds. The outbreaks of severe acute respiratory syndrome (SARS) (2003), Middle East Respiratory Syndrome (MERS) (2012), and the recent outbreak of COVID-19 (SARS-CoV-2) have shown the immense potential of these viruses to infect humans, causing deaths and large economic losses. Although the fatality rate of COVID-19 (~4%) is lower compared to SARS (~10%) and MERS (~35%), the rate of its spreading is much faster than both [1]. To date, more than 630 million people have been infected worldwide and more than 6.5 million people have already died. COVID-19 infection can cause symptoms ranging from mild cold-like symptoms to severe illness with pneumonia, respiratory problems, and death [2,3], Several countries have imposed lockdowns, which are helping in restricting the spread of the disease; however, it has not been completely successful. Besides loss of human lives, COVID-19 is causing severe economic losses to both developed and developing countries. Random mutations in this virus create new pathological strains [4]. To date, more than 40 new strains of coronavirus have been confined. The WHO classified these mutant strains as VOC (variants of concern) and VIC (variants of interest) as of 31 May 2021 (https://www.who.int/activities/tracking-SARS-CoV-2-variants) (accessed on 15 October 2022). Currently, VOC only contain different strains of Omicron variants. Additionally, these mutants have relatively slight resistance to the existing medications and vaccines. The best examples of how a change in just a few amino acids can change a virus’s basic nature are the Omicron and Delta variants, which were just recently discovered [5,6,7,8].

The coronavirus enters the cell with the help of its trimeric glycosylated spike (S) protein, a class I viral fusion protein with two subunits, S1 and S2. A fusion peptide is located between the *N*- and the C-terminal regions of S2 [9]. The S1 subunit binds with the angiotensin-converting enzyme-2 (Ace-2) followed by S2 domain and peptide-mediated fusion of the viral envelope and cell membrane [10]. Proteolytic cleavage of the S protein after binding with Ace-2 by various host cell proteases such as Transmembrane Serine Protease 2 (TMPRSS2), endosomal cathepsins, etc., causes the fusion of the viral envelope to the host cell and delivers the viral nucleocapsid into the host cell [11,12,13]. The released genomic RNA of the virus is then recognized by the host cell translation machinery, which synthesizes the polyprotein 1a (pp1a) and 1ab (pp1ab) by ribosomal frame shifting [14]. Then, the proteolytic cleavage of the polyproteins by main protease (M^pro^) produces various nonstructural proteins (nsp) [15]. It has been shown previously that the nsp5 (M^pro^) of porcine coronavirus can also modify the key players of the host immune system by mediating the cleavage of NFκB and STAT-2, therefore affecting the production of IFN-β and expression of interferon-stimulated genes [16].

Currently, there are many drug candidates in clinical use or undergoing clinical trials worldwide to treat COVID-19. For example, there are Paxlovid, which is a combination of two antiviral medicines named nirmatrelvir and ritonavir, Molnupiravir, Bebtelovimab, Tocilizumab, Baricitinib, Ensovibep, Sabizabulin, etc. Remdesivir, which was originally developed for the treatment of the Ebola outbreak, was given to a patient in the United States, causing the recovery of a patient from the severely ill category [17,18]. Limited clinical studies on two antimalarial drugs, chloroquine and hydroxychloroquine, have shown potential for treating COVID-19 [19]. However, the toxicity associated with the use of these drugs has also been reported [20]. Several vaccines for the novel coronavirus are available, and a few of them are also under clinical trials (https://www.cdc.gov/coronavirus/2019-ncov/vaccines/stay-up-to-date.html#:~:text=Four%20COVID%2D19%20vaccines%20are,Novavax) (accessed on 16 October 2022).

Coronavirus M^pro^ is a dimeric protein that cleaves the polyprotein into several functional proteins, helping in viral replication and transcription [21]. Hence, M^pro^ is an important drug target for treating COVID-19 [22]. M^pro^ is a three-domain protein comprising domains I, II, and III. Domains I and II have a chymotrypsin-like fold [21]. The substrate-binding cleft of M^pro^ is located between domains I and II. M^pro^ is not proteolytically active in the monomeric form because the substrate-binding site is not well-organized, while in the dimeric form, the substrate-binding site adopts proper conformation [15].

Here, using the Schrödinger software suite, we screened the FDA-approved drug library against SARS-CoV-2 M^pro^ to search for the drugs that can be potentially used for treating the COVID-19 pandemic. Our data suggest that several drugs could potentially bind and inhibit M^pro^ activity. Considering safety profile and bioavailability, drugs among the top hits that could be taken further for in vitro and in vivo studies include lumefantrine, dipyridamole, dihydroergotamine, hexoprenaline, and riboflavin as potential candidates for treating COVID.

## 2. Materials and Methods

### 2.1. Multiple Sequence Alignment and ConSurf Analysis

For multiple sequence alignment, M^pro^ sequences of SARS-CoV-2, SARS-CoV, MERS, human coronavirus NL63 (H-CoV), infectious bronchitis virus (IBV), porcine epidemic diarrhea virus (PEDV), and bat coronavirus were extracted from the NCBI database (www.ncbi.nlm.nih.gov) (accessed on 25 March 2020) and then aligned using the COBALT tool [4,23] using default settings. Then, a multiple sequence alignment file was submitted to the ESPRIPT 3.0 server [24] for rendering sequence similarity and secondary structure information from the aligned sequences. The PDB ID 6M03 of SARC-Cov-2 M^pro^, was used for secondary structure assignment. ConSurf analysis was performed to generate a conservation score on PDB ID 6M03 using the HMMER homology search algorithm with E-value 0.0001 and UNIREF-90 protein database, and multiple sequence alignment was generated using MAFFT. Fifty sequences sharing >60% sequence identity were used for ConSurf analysis [25].

### 2.2. FDA-Approved Small Molecule Library Preparation

The drug bank database (www.drugbank.ca) (accessed on 20 March 2020) was used to download the FDA-approved drug files, which were then prepared for docking studies using the LigPrep module of Schrödinger to produce energy-minimized 3D molecular structures [26]. Downloaded FDA drug compound databases contain only 2D molecular structures in SDF format. LigPrep efficiently and accurately performed 3D conversion of these FDA-approved drug compounds. Now, this 3D structure compound library can be utilized for docking studies.

### 2.3. Structure-Based In Silico Screening and Scoring

In the current study, for the M^pro^ structure that was solved with an inhibitor called N3, PDB ID 6LU7 was used as a receptor for docking [27]. The protein preparation wizard module of the Schrödinger software suite was used to prepare the M^pro^ structure. The prepared receptor structure was further analyzed by the SiteMap module of the Schrödinger software suite to identify potential binding sites. SiteMap ranks potential binding sites based on size, functionality, and the degree of solvent exposure on the protein using the site score function [28,29]. The predicted binding site with highest site map score (>0.9) was further selected for grid generation. The Receptor Grid Generation module was used to generate the docking grid with keeping the box dimension at 21 Å. The molecules were docked using Xtra- precision (XP) mode in the Glide module [30].

### 2.4. Binding Energies Calculation Using MM-GBSA

The MM-GBSA module of the Schrodinger software suite was used to determine the binding energies of M^pro^ with docked FDA-approved molecules [31]. Both the docked protein and the ligand complex were manually separated before being loaded in the MM- GBSA module. Five different energy calculation techniques are included in MM-GBSA analysis, including optimization of the free ligand, optimization of the free receptor, optimization of the complex, optimization of the ligand from the minimized complex, and receptor from the minimized complex [31].

### 2.5. Molecular Dynamics Simulations

The docked protein–drug were subjected to a system-builder panel of the Desmond module incorporated in the Schrodinger software suite [32]. The complexes were embedded in TIP-3P solvation system in an orthorhombic box with the buffer distance of a, b, c = 10 Å. The system was solvated by adding water molecules and NaCl concentration was kept at 150 mM. After building the solvent environment, the model system was relaxed with the default multistep Desmond relaxation protocol. The simulation was carried out at 300 K and 1.013 bar for 100 ns with trajectory-recording intervals of 20 ps and energy-recording intervals of 1.2 ps in the NPT ensemble class. The OPLS3 force field was used for MD simulations [33].

## 3. Results

### 3.1. Sequence and Structural Comparison of M^pro^

**Figure 1:**
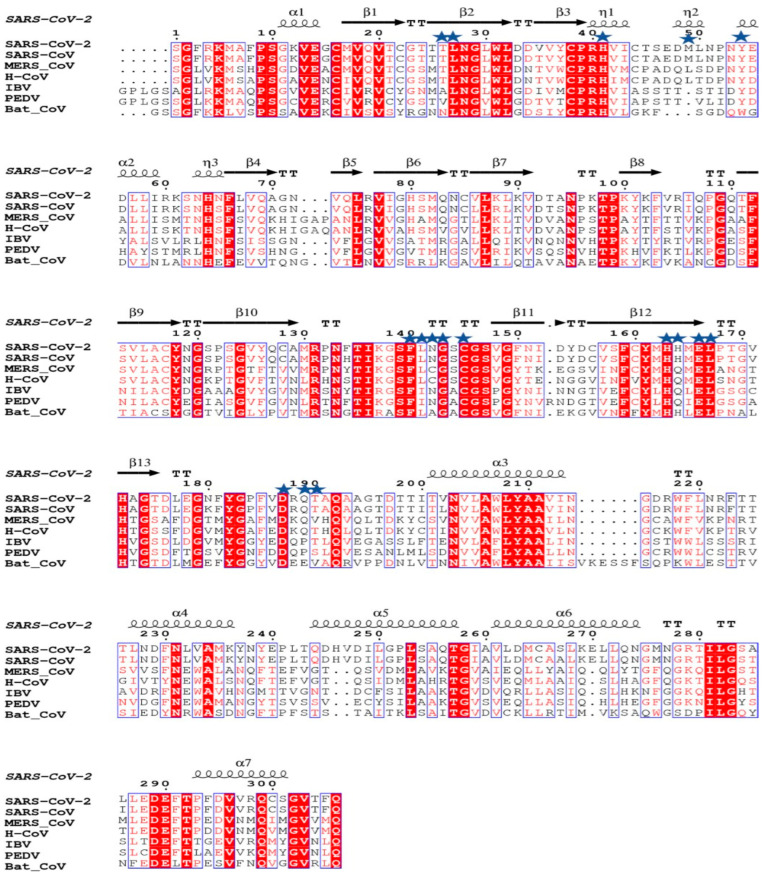
Multiple sequence alignment of M^pro^ of SARS-CoV-2 with M^pro^ of other coronaviruses performed using ESPRIPT 3.0 server. PDB ID 6M03 was used as a reference for assignment of secondary structural elements. The conserved residues involved in interaction with ligands are highlighted with blue stars. The substrate binding pocket of M^pro^ is formed by these conserved residues.

**Figure 2:**
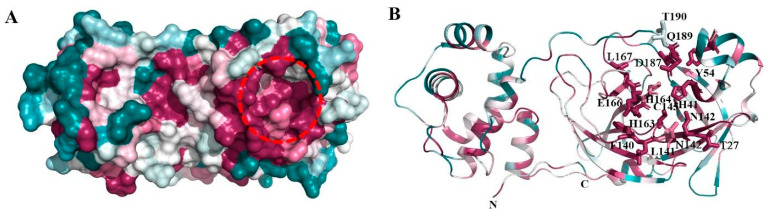
Consurf analysis of M^pro^ structure: ConSurf analysis showing conserved regions in the three-dimensional structure of M^pro^. (**A**) Surface view showing the highly conserved substrate-binding site (red broken circle) and region involved in homodimerization (broken blue circle). Drugs were docked in the substrate-binding region of M^pro^. (**B**) Cartoon representation showing the conserved regions in M^pro^. The residues forming the substrate-binding pocket are shown in stick representation.

The structural analysis of the protein inhibitor complex (PDB ID: 6LU7) revealed that covalent inhibitor N3 binds at the hydrophobic ligand binding pocket and interacts with His163, His164, Glu166, Gln189, and Thr190 residues of M^pro^ [27]. Multiple sequence alignment and ConSurf analyses suggest that this ligand binding site is highly conserved among the members of the coronavirus family (Figure 1 and Figure 2) [25]. In MERS and H-CoV, the N142C mutation was observed, while in MERS, H-CoV, IBV, and PEDV, the H164Q mutation was observed. Additionally, in MERS and Bat-CoV, the T191V mutation was observed. At the position 215, Bat-CoV has a stretch of residues, i.e., VKESSF, which is absent in all other coronaviruses. We also observed insertion of H247, V248, and E270 in both SARS-CoV-2 and SARS-CoV. These residues are absent in the other coronaviruses analyzed in this study (Figure 1).

### 3.2. In Silico Screening of FDA-Approved Drugs to Identify Potential Binders

**Figure 3:**
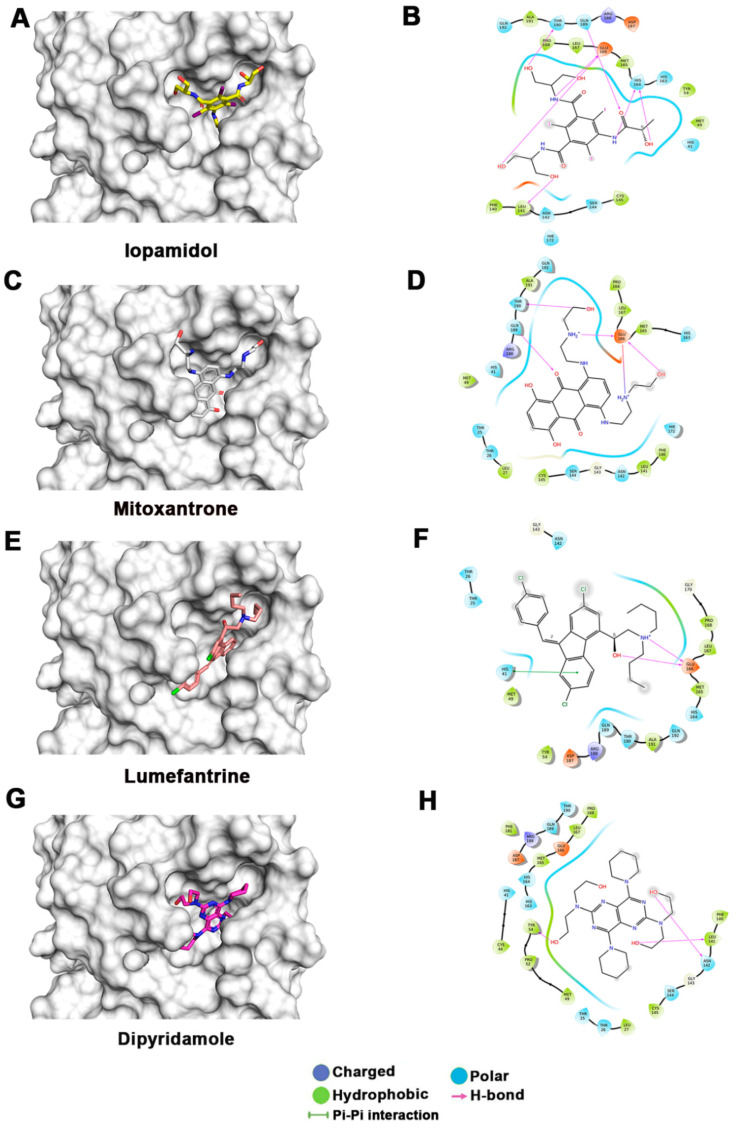

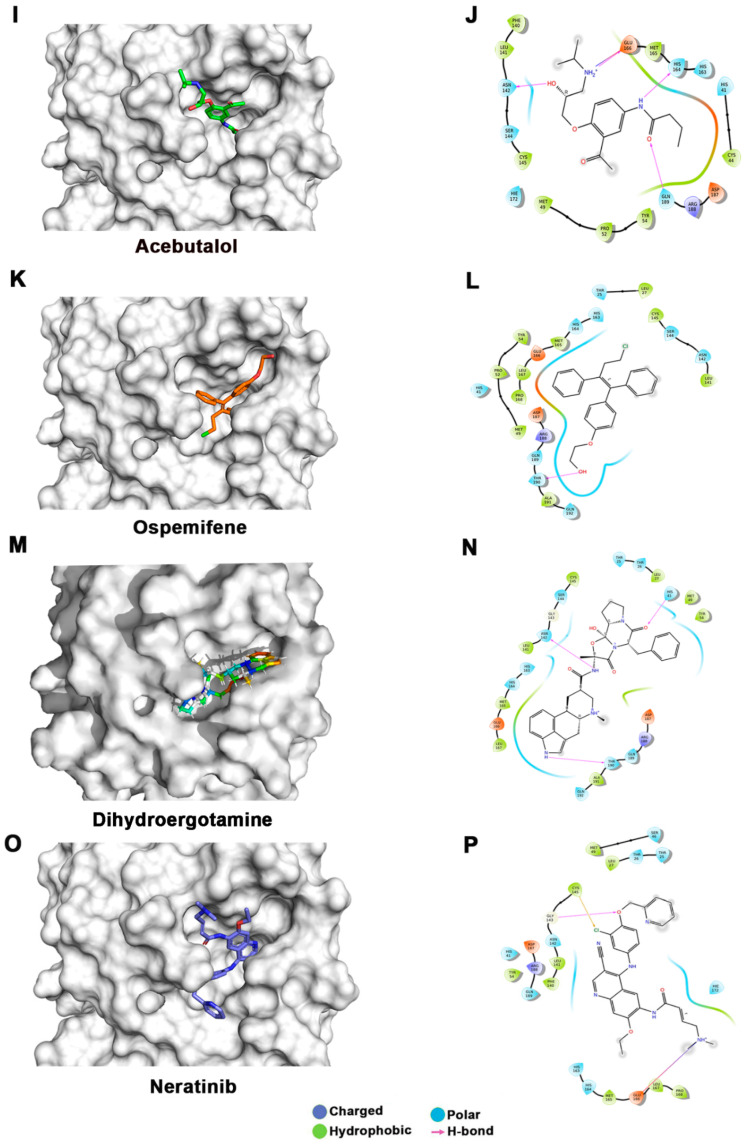

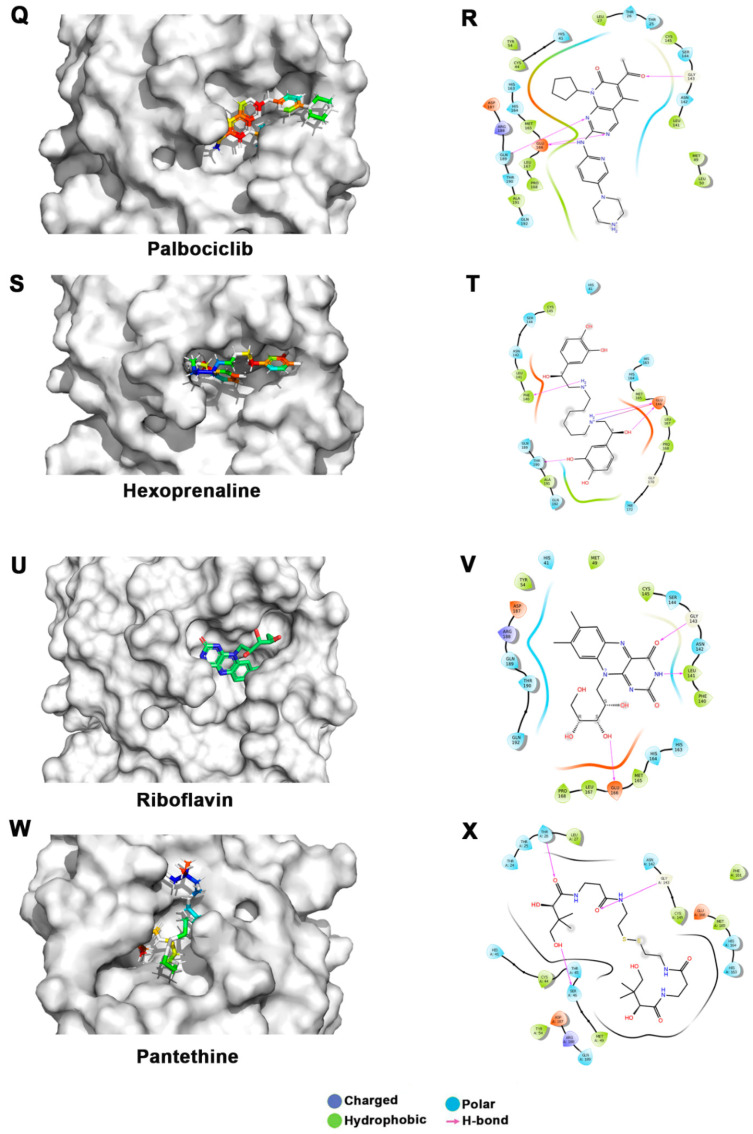
Docking studies of FDA-approved drugs on M^pro^ structures: Docking of FDA-approved drugs (shown in stick representation) (**A**) Iopamidol, (**C**) Mitoxantrone, (**E**) Lumefantrine, (**G**) Dipyridamole, (**I**) Acebutalol, (**K**) Ospemifene, (**M**) Dihydroergotamine, (**O**) Neratinib, (**Q**) Palbociclib, (**S**) Hexoprenaline, (**U**) Riboflavin, and (**W**) Pentethine in the substrate-binding pocket of M^pro^ (Grey surface representation). Carbon, nitrogen, and oxygen are shown in green, blue, and red colors, respectively. (**B**) A 2D interaction map of drugs, with M^pro^ highlighting various interactions stabilizing protein–drug interactions. (**B**) Iopamidol, (**D**) Mitoxantrone, (**F**) Lumefantrine, (**H**) Dipyridamole, (**J**) Acebutalol, (**L**) Ospemifene, (**N**) Dihydroergotamine, (**P**) Neratinib, (**R**) Palbociclib, (**T**) Hexoprenaline, (**V**) Riboflavin, and (**X**) Pentethine.

The receptor and the FDA-approved drug library preparation for molecular docking studies were performed using Schrödinger Suite, 2019, using the protein preparation wizard and LigPrep modules [26]. The potential sites for drug binding in the crystal structure were discovered using the SiteMap tool [28]. Five potential druggable sites for ligand binding were identified by SiteMap analysis, with site scores ranging from 0.549 to 0.943. Grid generation and docking were both performed on the top binding site, which had a site score of 0.943. This site corresponds to the highly conserved substrate-binding pocket in coronaviruses. The molecular docking was carried out using the Glide Xtra-Precision (XP) module, which use the E-model scoring function, in combination with the anchor-and-grow method for sampling, to select between protein–ligand complexes of a given ligand and the GlideScore [34]. The generalized born and surface area continuum solvation (MM-GBSA) module and the molecular mechanics energies were also used to perform binding-free-energy calculations on the XP-docked structures [31]. We shortlisted 50 potential drugs from the FDA-approved library based upon both XP docking energy and MM-GBSA scores (Table 1). Molecular docking of >2200 FDA-approved drugs at the similar hydrophobic core resulted in more than 50 potential drug molecules having a docking score of <5.0 and an MM-GBSA score of <30 (Table 1). The docking and MM-GBSA scores imply that these drugs may interact favorably, which might possibly inhibit M^pro^ activity. Figure 3 displays the top docking pose and ligand–protein interactions for the top 12 hits.

The lowest docking score and MM-GBSA-based binding energy of −9.93 Kcal/mol and −77.27 Kcal/mol, respectively, were observed for iopamidol. Iopamidol interacts with the substrate-binding site by forming hydrogen bonds with Leu141, His164, Glu166, Gln189, and Thr190 and several nonbonded interactions. Iopamidol is a radiopaque contrast agent that contains iodine and is used for imaging of organs, blood vessels, and other tissues on a CT scan or other radiologic (X-ray) examination [35]. Like iopamidol, another radiocontrast agent, metrizamide, was also among the top drugs in our list [36,37]. Another top molecule in our list was mitoxantrone, having a docking score of −8.3 Kcal/mol and an MM-GBSA score of −69.9 Kcal/mol. Binding of mitoxantrone is mediated by several nonbonded interactions and a network of hydrogen bonds with Glu166, Thr190, and Gln189. Mitoxantrone is an immune suppressor and anticancer agent used for treating multiple sclerosis and cancer [38,39,40]. Interestingly, this drug has also been reported as a potential M^pro^ binder by Zhang et al. [41].

Lumefantrine, with a docking score of −5.136 Kcal/mol and an MM-GBSA score of –66.8 Kcal/mol, is at the third position in our list. It binds M^pro^ with several nonbonded interactions, forms a hydrogen bond with Glu166, and has a π–π interaction with His41. Lumefantrine is an antimalarial agent used in treating acute uncomplicated malaria [42]. The next drug in our list is dipyridamole, which is a phosphodiesterase-2 inhibitor that blocks the metabolism and uptake of adenosine by erythrocytes and vascular endothelial cells [43]. It causes blood vessel dilation and inhibits blood clot formation and is used as a coronary vasodilator [43]. Docking analysis suggested that dipyridamole forms both hydrophobic and hydrophilic interactions with M^pro^ and interacts with Tyr54, Asn142, and Leu141 through hydrogen bonding, and its docking and MM-GBSA scores are −7.2 Kcal/mol and −65.56 Kcal/mol, respectively. Acebutolol is another potential drug that showed favorable binding in our docking studies, having a docking score of −7.3 Kcal/mol and an MM-GBSA score of −61.42 Kcal/mol. Docking analysis suggested that acebutolol is stabilized by hydrophobic interactions and hydrogen bonding with Asn142, His164, Glu166, and Gln189 residues in the M^pro^ binding pocket. Acebutolol is a cardio-selective, β-adrenoreceptor blocking agent. Acebutolol is used for treating high blood pressure, and it reduces an irregular heartbeat [44]. Ospemifene is a nonhormonal estrogen-receptor modulator that is used for the treatment of dyspareunia [45,46]. This drug also forms hydrophobic interactions and hydrogen bonding and interacts with Thr190 in the binding pocket, with −6.6 Kcal/mol docking and −60.68 Kcal/mol MM-GBSA scores. The next drug in this list is dihydroergotamine, a derivative of ergotamine and mainly used for the treatment of acute migraine. It docks with a binding score of −6.3 Kcal/mol and an MM-GBSA score of −60.62 Kcal/mol. Besides hydrophobic interactions, it forms hydrogen bonds with His 41, Thr190, and Asn142. Dihydroergotamine can be administered as a nasal spray; therefore, it can potentially be effective against pulmonary indications of COVID-19 infection [47]. Neratinib and palbociclib are two other anticancer drugs that appeared in our list which are used to treat early-stage HER2-positive or HER2-negative breast cancer patients, respectively [48,49]. Neratinib is known to limit the development of cancer cells by blocking their tyrosine kinase. It forms a hydrogen bond with Cys145, Cys189, Gly143, and Glu 166 residue with M^pro^ protein, and its docking and MM-GBSA scores are −6.56 Kcal/mol and −59.57 Kcal/mol, respectively. Another effective drug in our top-ten list is palbociclib. It is a cancer-preventive drug that inhibits cyclin-dependent kinases. It forms a hydrogen bond with Gln189, Glu 166, and Gly 143 residue with M^pro^ protein, and its docking and MM-GBSA scores are −6.29 Kcal/mol and −59.51 Kcal/mol, respectively.

Hexoprenaline, which functions as a bronchodilator and an antiasthmatic and tocolytic agent by stimulating β-2 adrenergic receptors, is among the top-10 compounds in the list [50]. Besides several nonbonded interactions, hexoprenaline forms hydrogen bonds with Phe140, Glu166, and Thr190. Its binding and MM-GBSA scores are −6.35 and −58.35, respectively.

Interestingly, in our docking studies, we also found two water-soluble vitamins, Riboflavin and Pantethine (derivative of vitamin B5), to be potential binders of M^pro^. Riboflavin (Vitamin B2) is at the fifteenth position in our list. It binds with docking and MM-GBSA scores of −7.29 Kcal/mol and −53.66 Kcal/mol, respectively. It forms a hydrogen bond with Leu141, Gly143, and Glu166 in addition to having several nonbonded interactions. Pantethine appeared at the thirty-second position in the list, and it is a dimeric form of pantetheine, which is produced from pantothenic acid by the addition of cysteamine. It works as a cholesterol-lowering drug. It binds with docking and MM-GBSA scores of −6.365 Kcal/mol and −40.82 Kcal/mol, respectively. It forms a hydrogen bond with Thr26, Ser46, and Gly143 residues. Both riboflavin and pantethine, though having relatively lower ranks based on docking and MM-GBSA scores, being the safest biomolecules among the list, can be taken forward to evaluate M^pro^-inhibitory activities. The bioavailability, cell penetration, half-life, and safety profile make riboflavin and pantethine potential candidates to be screened as an inhibitor of M^pro^ (Figure 3).

### 3.3. Molecular Dynamics (MD) Simulations

**Figure 4:**
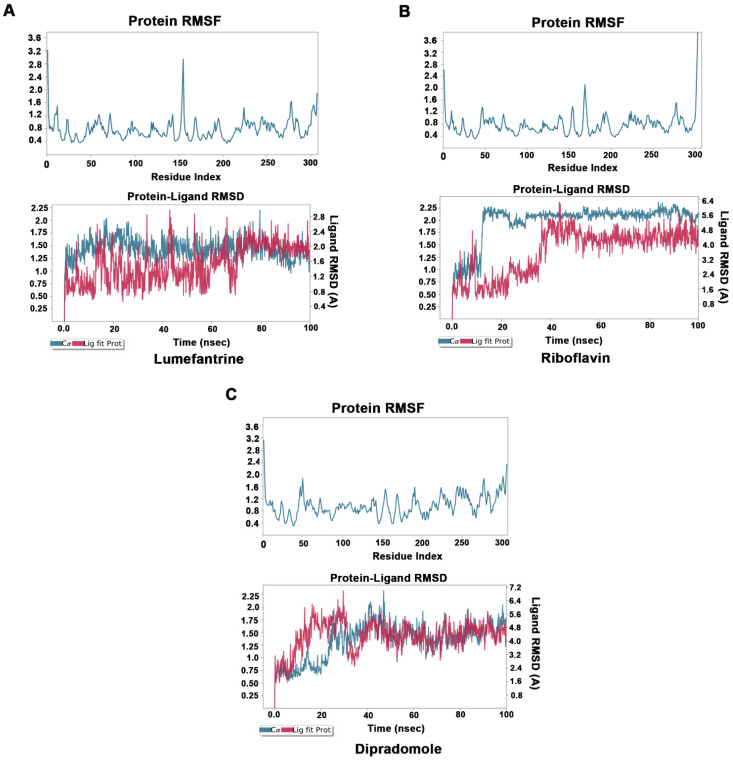
Molecular dynamics studies of M^pro^–drug complexes: Molecular dynamics simulations for M^pro^–lumefantrine (**A**), M^pro^–riboflavin (**B**), and M^pro^–dipradomole (**C**) complex. Low root mean square fluctuations of Cα protein atoms in upper panel suggest protein was stable during the simulation run. The stable RMSD of Cα protein atoms (Blue) and Ligand RMSD (Red) in lower panel suggests formation of stable protein–ligand complex.

To understand the dynamics of M^pro^–drug interactions, we selected M^pro^–lumefantrine, M^pro^–riboflavin, and M^pro^–dipradomole complexes for MD simulation studies. The stability and the fluctuations of the M^pro^–drug complex structures were analyzed by the RMSD and the RMSF plots of the ligands and protein Cα. The convergence of RMSD trajectory of M^pro^–drug complexes during 100 ns MD simulations suggests that the complexes are stable, and the ligand was bound favorably to the substrate-binding pocket. The average changes occurring in the protein backbone, as inferred from RMSD plots, suggest that the protein is stable and does not undergo overall major fluctuations (Figure 4A–C).

## 4. Discussion

SARS-CoV-2 caused the COVID-19 outbreak and resulted in enormous losses both in terms of economic output and human life, pushing research into viable therapies and cures. We employed drug repurposing and in silico structure-based drug-design techniques to look for such possible therapeutic agents. Several groups have targeted M^pro^ for developing effective drugs against SARS-CoV-2 using FDA drug libraries. Yuce et al. used FDA drug libraries and found Dihydroergotamine, which is also in our top-12 list of inhibitors molecules [51]. Balakrishnan et al. used Schrodinger software and compared the FDA-approved antiviral drugs’ efficiency against COVID-19 [52], and Molavi et al. used FDA-approved drugs to find new RdRp and CLpro inhibitors against the novel coronavirus [53].Yang et al. designed an effective inhibitor, N3, which binds at the substrate-binding site and inactivates SARS-CoV [54]. Recently, using a computer-aided drug-design strategy, Jin et al. also reported N3 to be a mechanism-based inhibitor of SARS-CoV-2 [27]. Khaerunnisa et al. implemented repurposing strategies to find out possible potential inhibitors against M^pro^. They reported nelfinavir and lopinavir to be potential inhibitors of M^pro^ [19]. Jin et al. reported Ebselen to exhibit excellent inhibitory activity against M^pro^, with an IC50 0.67 μM [27].

In our study, two radio contrast agents (iopamidol and iopromide) were among the top hits. However, these drugs have multiple side effects, ranging from itching to a life-threatening emergency known as contrast-induced nephropathy [55]. Therefore, these may not be suitable candidates for treating COVID-19. Similarly, there are three anticancer drugs (palbociclib, mitoxantrone, and neratinib) in the top hits which may have undesirable side effects; hence, they may not be suitable drug candidates. Lumefantrine, one of the top drugs in our list, has limited side effects and a longer half-life (three to six days), which makes it a potential drug candidate that may be taken up for further studies to test efficacy in inhibiting M^pro^ and, hence, in treating COVID-19. It binds M^pro^ with several nonbonded interactions, forms a hydrogen bond with Glu166, and has a π–π interaction with His41. Besides these, dipyridamole, dihydroergotamine, and hexoprenaline are other drugs on the top of the list and have good bioavailability and less toxicity. Dipyridamole is a well-established drug which is used as a coronary vasodilator. Docking analysis showed dipyridamole forms both hydrophobic and hydrophilic interactions with M^pro^ and interacts with Tyr54, Asn142, and Leu141 through hydrogen bonding. Dihydroergotamine has an added advantage over other drugs, as it can be administered as a nasal spray; hence, it can readily target the site of infection, i.e., lungs. It forms hydrogen bonds with His 41, Thr190, and Asn142. Hexoprenaline is a bronchodilator and an antiasthmatic and tocolytic agent which functions by stimulating β-2 adrenergic receptors, and it is among the top-10 compounds in the list [50]. Besides several nonbonded interactions, hexoprenaline forms hydrogen bonds with Phe140, Glu166, and Thr190. Using molecular docking studies, Narayanan et al. reported vitamin B12 to be a potential inhibitor of RNA-dependent-RNA polymerase [56]. So, the combination of vitamins B2, B5, and B12 may also be explored as potential treatment for COVID-19 that may exploit distinct mechanisms for inhibiting viral replication. Similarly, riboflavin, which is vitamin B2, showed promising results in docking and MD simulation studies. It forms a hydrogen bond with Leu141, Gly143, and Glu166, in addition to having several nonbonded interactions. It showed binding with M_pro_ in MD simulations up to 100 ns.

To conclude, among several hits obtained in our study, lumefantrine, dipyridamole, and riboflavin appear to be very potential candidates with good docking scores and stable RMSD/RMSF in MD simulations up to 100 ns (Table 1, Figure 4). In addition, dihydroergotamine, hexoprenaline, and pantethine also appeared to be attractive candidates as potential inhibitors of M^pro^. However, in vitro and in vivo studies are necessary to further investigate their therapeutic potential in treating COVID-19 or other related coronavirus infections.

## Figures and Tables

**Table 1:** List of top-51 hits with their respective docking and MM-GBSA scores and current clinical use of the drugs.

S. No.	Drug	Docking Score (Kcal/mol)	MM-GBSA Score (Kcal/mol)	Clinical Use
1	Iopamidol	−9.93	−72.77	Radiocontrast agent
2	Mitoxantrone	−8.354	−69.9	Anticancer
3	Lumefantrine	−5.136	−66.8	Antimalarial
4	Dipyridamole	−7.193	−65.56	Vasodilator
5	Acebutolol	−7.397	−61.42	Antiarrhythmia
6	Ospemifene	−6.58	−60.68	Estrogen receptor modulator
7	Dihydroergotamine	−6.301	−60.62	Vasoconstrictor
8	Neratinib	−6.564	−59.57	Anticancer
9	Palbociclib	−6.297	−59.51	Anticancer
10	Hexoprenaline	−6.352	−58.35	Antiasthmatic
11	Prazepam	−6.28	−57.77	Anxiolytic, sedative
12	Dipivefrin	−6.254	−57.56	Antiglaucoma
13	Doxorubicin	−6.217	−56.45	Anticancer
14	Rosuvastatin	−6.443	−53.93	Antiobesity
15	Riboflavin	−7.219	−53.66	Vitamin B2
16	Iopromide	−7.392	−51.78	Radiocontrast agent
17	Afatinib	−6.822	−49.6	Anticancer
18	Fluvastatin	−6.628	−49.45	Antiobesity
19	Metrizamide	−8.449	−49.24	Radiocontrast agent
20	Pitavastatin	−6.468	−49.17	Antiobesity
21	Talniflumate	−6.763	−48.31	Anti-inflammatory
22	Lucanthone	−6.741	−47.93	Schistosomicides
23	Prezatide	−7.183	−47.55	Wound healing
24	Fluvoxamine	−6.348	−46.46	Antidepressant
25	Canagliflozin	−6.469	−46.44	Ant-diabetic
26	Xanthinol	−6.981	−45.04	Vasodilator
27	Pravastatin	−6.283	−44.31	Antihypercholesterolemia
28	Fominoben	−6.093	−44.31	Antitussive
29	Esculin	−6.25	−44.28	Antioxidant
30	Imipenem	−6.979	−42.5	Antibiotic
31	Betaxolol	−6.625	−41.1	Antihypertension
32	Pantethine	−6.365	−40.82	Vitamin B5
33	Benzylpenicilloyl polylysine	−6.438	−40.41	Antihistamine
34	Arbutin	−6.647	−39.09	Skin lightening, antimelanin
35	Iron saccharate	−7.018	−38.87	Iron deficiency anemia treatment

## Data Availability

Not applicable.
